# An Unclassified Microorganism: Novel Pathogen Candidate Lurking in Human Airways

**DOI:** 10.1371/journal.pone.0103646

**Published:** 2014-07-31

**Authors:** Kazumasa Fukuda, Kazuhiro Yatera, Midori Ogawa, Toshinori Kawanami, Kei Yamasaki, Shingo Noguchi, Robert S. Murphy, Hiroshi Mukae, Hatsumi Taniguchi

**Affiliations:** 1 Department of Microbiology, University of Occupational and Environmental Health, Japan, Kitakyushu, Fukuoka, Japan; 2 Department of Respiratory Medicine, University of Occupational and Environmental Health, Japan, Kitakyushu, Fukuoka, Japan; 3 Center of Fundamental Education, University of Kitakyushu, Kitakyushu, Fukuoka, Japan; University of Lausanne, Switzerland

## Abstract

During the assessments of the correlation of the diseases and the microbiota of various clinical specimens, unique 16S ribosomal RNA (rRNA) gene sequences (less than 80% similarity to known bacterial type strains) were predominantly detected in a bronchoalveolar lavage fluid (BALF) specimen from a patient with chronic lower respiratory tract infection. The origin of this unique sequence is suspected to be the causative agent of the infection. We temporarily named the owner organism of this sequence “IOLA” (Infectious Organism Lurking in Airways). In order to evaluate the significance of IOLA in human lung disorders, we performed several experiments. IOLA-16S rRNA genes were detected in 6 of 386 clone libraries constructed from clinical specimens of patients with respiratory diseases (in our study series). The gene sequences (1,427 bp) are identical, and no significantly similar sequence was found in public databases (using NCBI blastn) except for the 8 shorter sequences detected from patients with respiratory diseases in other studies from 2 other countries. Phylogenetic analyses revealed that the 16S rRNA gene of IOLA is more closely related to eukaryotic mitochondria than bacteria. However, the size and shape of IOLA seen by fluorescent *in-situ* hybridization are similar to small bacteria (approximately 1 µm with a spherical shape). Furthermore, features of both bacteria and mitochondria were observed in the genomic fragment (about 19 kb) of IOLA, and the GC ratio of the sequence was extremely low (20.5%). Two main conclusions were reached: (1) IOLA is a novel bacteria-like microorganism that, interestingly, possesses features of eukaryotic mitochondria. (2) IOLA is a novel pathogen candidate, and it may be the causative agent of human lung or airway disease. IOLA exists in BALF specimens from patients with remarkable symptoms; this information is an important piece for helping solve the elusive etiology of chronic respiratory disorders.

## Introduction

The development and prevalence of PCR and DNA sequencing technologies [1] has expanded microbiological studies in various environments harboring vast microbial diversity that cannot be covered by culture-methods. The studies based on the molecular methods to detect pathogenic microbes using clinical specimens have also been vigorously conducted [2]. However, the discoveries of new bacteria showing novelty at a high classification level have been more remarkable from environmental samples than from clinical samples. In order to evaluate the correlation of the diseases and the microbiota and also discover new pathogens, we analyzed the bacterial communities based on the 16S rRNA gene sequence of various clinical specimens [3]–[6]. Contrary to our expectations, most of the 16S rRNA gene sequences detected in the clinical specimens showed high similarity with known bacterial species. However, during the process of the microbiota analyses, unique 16S rRNA gene sequences (less than 80% similarity to known bacterial type strains) were predominantly detected in a BALF specimen from a patient with chronic lower respiratory tract infection. The origin of this unique sequence was suspected to be the causative agent of the infection. We temporarily named the organism IOLA for “Infectious Organism Lurking in human Airways”.

Recently, studies applying next-generation sequencing to research the human lung microbiome have been increasing rapidly [7]–[11]. Investigations to clarify the microbial communities of various types of specimens from healthy people and diseased people have shown remarkable growth in recent years, especially with chronic respiratory diseases such as asthma [12], chronic obstructive pulmonary disease (COPD) [13]–[15], and cystic fibrosis (CF) [16]–[18], which were originally recognized as highly heterogeneous diseases. For example, many new findings have been published regarding, but not limited to: (1) the differences in airway microbial composition and quantities between healthy subjects and those with the obstructive lung diseases, (2) the effects of treatments with antibiotics or steroids for microbial communities in patients, and (3) the effects of oral bacteria on the microbiome of human lung specimens. Though these studies revealed valuable evidence, there have been no reports concerning the discovery of novel pathogens (at a high taxonomic level) in human lung and/or airway specimens. The detection of novel microorganisms such as IOLA is extremely rare. The etiology of many chronic respiratory disease cases thus far has been incomplete. The clarification of IOLA’s properties is expected to be a significant component for a more complete etiology of such diseases. We performed several experiments using the particular BALF specimens to find further clues about this organism.

## Results

### Detection of IOLA 16S rRNA genes

**Figure 1:**
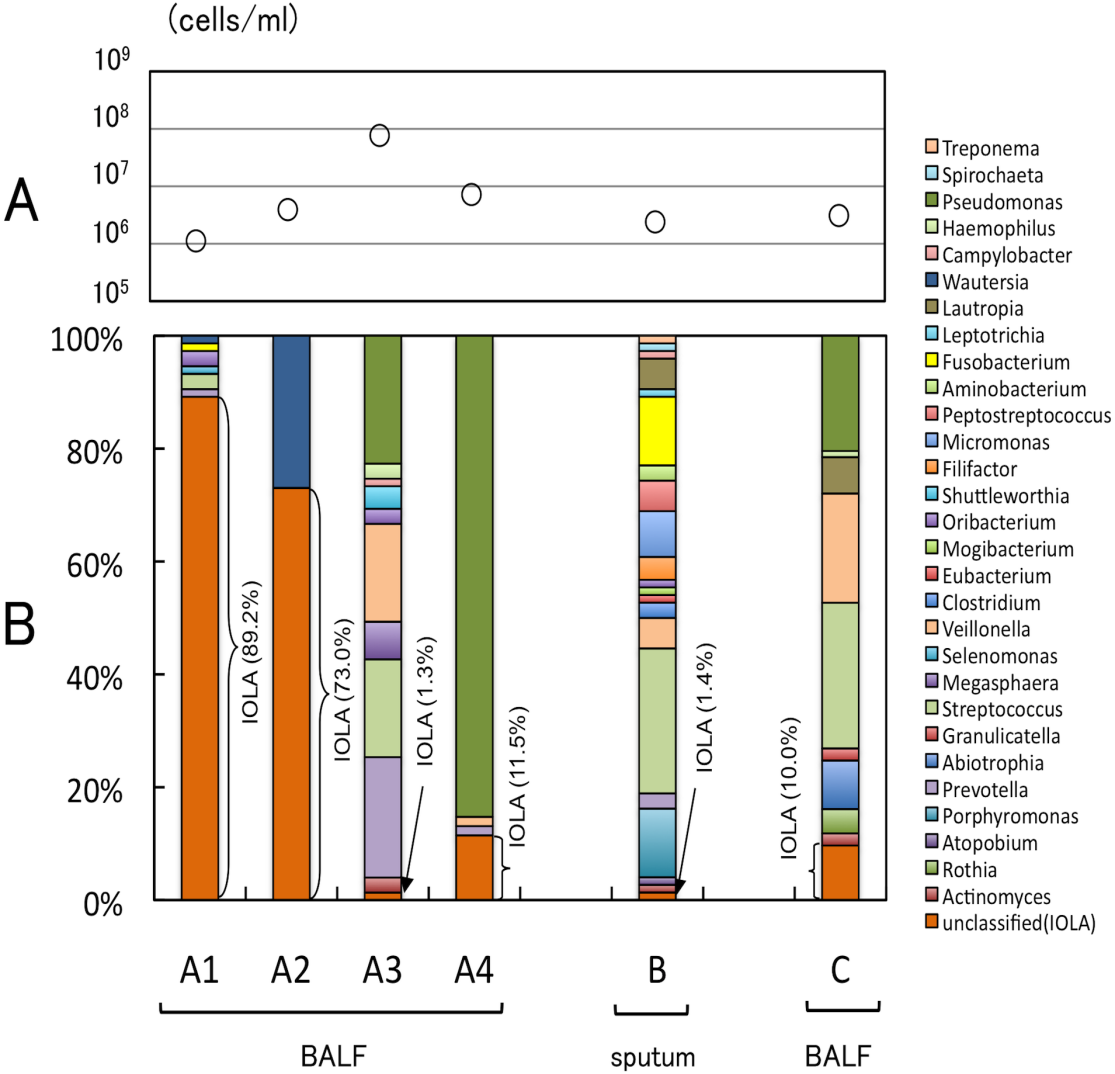
Bacterial cell numbers and compositions in the specimens from patients, detected IOLA in clone library analysis. **A,** Results of bacterial cell counts of the specimens from patient A using an epifluorescent staining method with ethidium bromid. Open circles indicate numbers of bacterial cells per ml of each the specimens. **B,** Percentage of the detected bacteria in the specimens with the clone library analysis of 16S rRNA gene. The percentages of IOLA-clones (orange box) in each of the clone libraries are shown in parentheses.

Unique 16S rRNA gene sequences were detected in 6 of 386 clone libraries constructed from clinical specimens of patients with respiratory diseases (in our study series from May 2010 through February 2013). Four of the 6 specimens were obtained from the same patient (patient A), and the other 2 specimens were from 2 patients (patients B and C). All of the 3 patients showed symptoms of lower respiratory tract infections (Table 1). As the first case in our study series, a novel clone (IOLA-clone) was predominantly detected (66/74 clones; 89%) as the result of clone library analysis of the BALF specimen (A1) obtained from patient A (Figure 1). After this episode, the patient was hospitalized 3 more times due to complications of lower respiratory tract infections. The IOLA-clone was also predominantly detected in the second specimen (A2) (Figure 1). The composition of the clone decreased in the next 2 specimens (A3 and A4) and showed a relative increase of *Pseudomonas* spp. The IOLA-clone was also detected in sputum of patient B1 (1 of 74 clones 1.4%) and in BALF of patient C1 (9 of 90 clones 10%) (Figure 1). The bacterial cell numbers in all of the specimens from the patients were over 10^6^ cells/ml (Figure 1). At least in the A1 and A2 cases, the high detection ratios of IOLA clones, the bacterial cell numbers, and the clinical findings (such as white blood cell counts, C-reactive protein, and observation of nodular shadows by chest computed tomography), suggested that IOLA may play a role in the symptoms (Figure 1 and Table 1).

**Table 1:** Patient characteristics.

Patients	A				B	C
Sampling(date)	2010.9.8	2011.9.2	2011.11.5	2011.12.24	2011.7.27	2012.6.1
Sample	A1	A2	A3	A4	B1	C1
Age (yr)	69	70	70	71	74	42
sex	F	F	F	F	M	M
Diagnosis	Bronchiectasis and Bacterialpneumonia	Bronchiectasisand Bacterialpneumonia	Bronchiectasisand Bacterialpneumonia	Bronchiectasis and Bacterial pneumonia	Bacterialpneumonia	Bacterial pneumonia and Interstitial pneumonia
Body temp.(°C)	37	37.2	38.4	36.8	38.5	36.7
aWBC(cells/µl)	9,600	13,400	12,899	12,710	11,200	4,600
bCRP(mg/dl)	6.6	1.7	18.0	7.2	15.6	2.0
Chest cCTfindings	Bronchiectasis,nodular shadows(bilateral)	Bronchiectasis,nodular shadows(bilateral)	Bronchiectasis, Deteriorationof nodularshadows(bilateral)	Bronchiectasis, Deteriorationof nodular shadows (bilateral)	Consolidation(left lowerlobe)	Consolidation(left lowerlobe)
Antimicrobial treatment	No medication	dGRNX	eMEPM	eMEPM	eMEPM	eMEPM
Therapeutic effect	fN/A	Effective	Effective	Effective	Effective	Effective

a WBC; white blood cell,

b CRP; C-reactive protein,

c CT; computed tomography,

d GRNX; Garenoxacin,

e MEPM; Meropenem,

f N/A; not applicable.

### Culture examinations

In an attempt to culture IOLA, A4 BALF specimen was cultured using 12 kinds of agar plates, a liquid medium, and a semisolid medium (Table S1). Moreover, infection experiments using J774 (murine macrophage-like) and A549 (human alveolar adenocarcinoma) cell lines were also performed. Many bacterial colonies and bacterial growth were observed under these culture conditions (except for infection experiments), however, multiplication of IOLA was not detected by using the IOLA-specific PCR examinations (see material and method section) in this study.

### Analyses of 16S rRNA gene sequences

The four 16S rRNA gene sequences (1,427 bp) of IOLA detected (Figure S1) from A1, A4, B1, and C1 (accession No. AB828319, AB828320, AB828321, and AB828322) were completely identical. The homology results from NCBI Nucleotide BLAST (blastn) [19] showed that there is no 16S rRNA gene sequence of known bacteria similar to the IOLA-sequences. However, the results also showed that 8 nucleotide sequences (shorter than our results) were similar to the sequence of IOLA with more than 96% similarity. Interestingly, the top 6 of 8 sequences (approximately 770 bp) (AY806120, AY806122, AY806119, AY806116, DQ188268, and DQ188269) were registered in the USA, and were detected in BALF of children with CF [20]. Another research group of the UK registered the other 2 sequences (approximately 550 bp) (GQ363263 and GQ364310). They were obtained from BALF specimens in patients with bronchial asthma or COPD [12]. All sequences in the NCBI nucleotide collection database (except for these 8 sequences) showed lower than 77% similarity with the IOLA sequence. In addition, we searched for IOLA-like sequences in the NCBI sequence read archive database, composing raw meta-genomic DNA sequences. From 2 meta-genomic datasets (ERX122937 and ERX122912), which were sampled from throat swabs of infants (healthy and non-infectious wheezing infants) in a large-scale project (ERP001558), a total of 8 sequences similar to the IOLA sequence with 97% similarity were detected. The detection ratios of the sequences in each dataset were 0.02% (4 of 16,995 reads) for ERX122937 and 0.2% (4 of 1,602 reads) for ERX122912, respectively. All sequences (except for these 8 sequences) of the 196 meta-genomic datasets (including 9,742,618 reads) relating to human lung or airway showed lower than 75% similarity to the IOLA sequence.

Furthermore, the IOLA sequence was analyzed with RDP Classifier software [21] and attained the following results: Domain; *Bacteria* (100%), Phylum; *Proteobacteria* (38%), Class; *Alphaproteobacteria* (25%); Order; *Rickettsiales* (22%), Family; *Rickettsiaceae* (21%), Genus; *Orientia* (21%). According to the results, IOLA was classified as a novel bacterium at the Phylum level.

**Figure 2:**
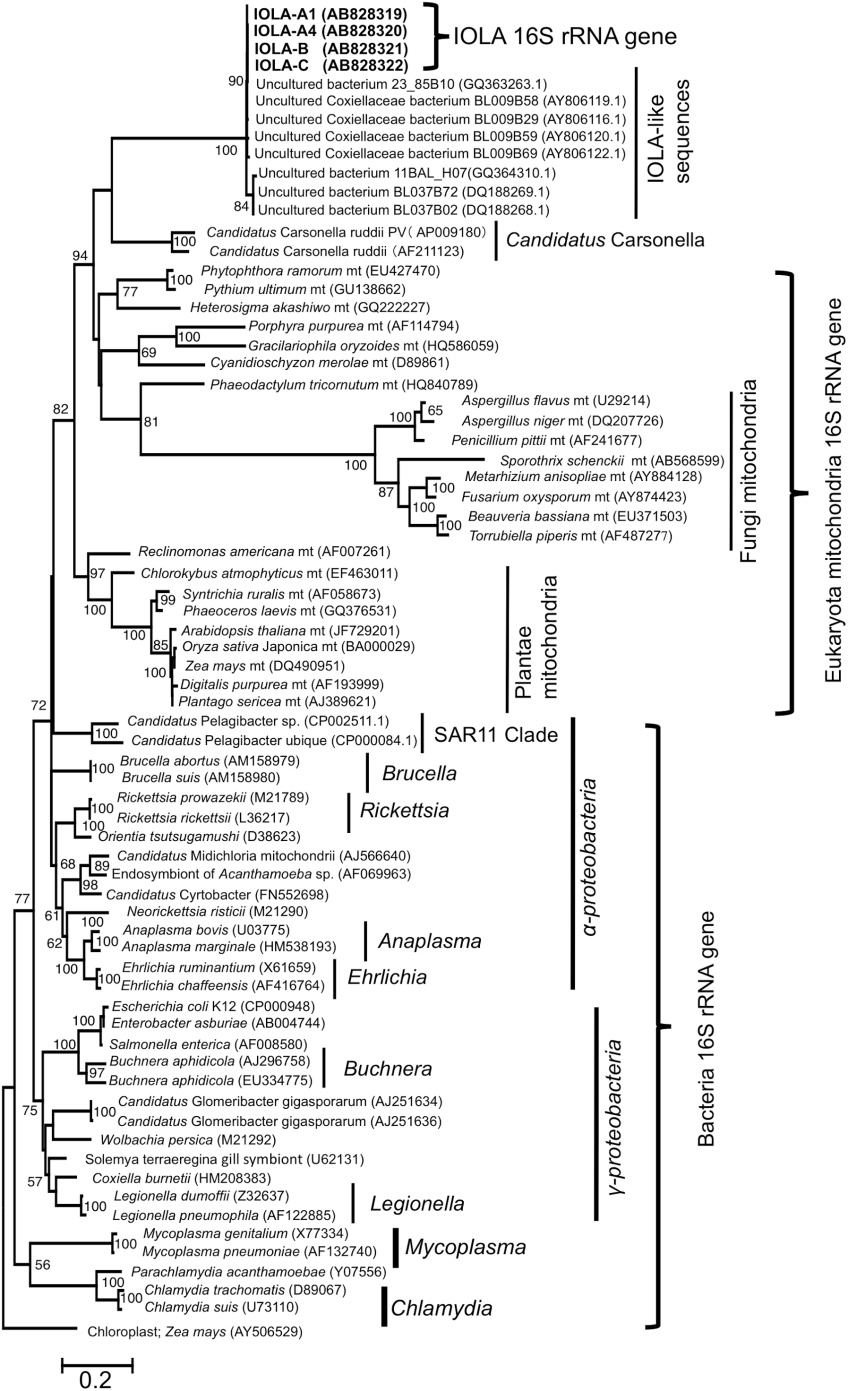
Maximum likelihood phylogenetic tree based on 16S rRNA gene sequences of bacteria and mitochondria of eukaryota. The phylogenetic tree was calculated with MEGA5.2.2 using the maximum likelihood method. The tree is based on the alignment of the 71 sequences (16S rRNA gene sequences). The percentages of bootstrap values for 1,000 replications are shown at the branching points (values less than 50% were ignored). The scale bar indicates substitutions per site. A sequence of chloroplast of *Zea mays* was used as an outgroup. Accession numbers of each sequence are shown in parentheses.

To dig deeper, we attempted a phylogenetic analysis with ARB-software [22] and the SSU Ref NR 111 dataset [23]. The result showed that the IOLA genes were located in the “mitochondria” cluster composed of mitochondrial 16S rRNA genes from Eukaryota, and was classified in *Rickettsiales* (order) of *Alphaproteobacteria* (class) on the phylogenetic tree. To confirm the taxonomical information, two more phylogenetic trees were constructed using maximum likelihood (ML) and neighbor-joining methods with ClustalW [24] and MEGA5.2.2 [25]. Both of the refined phylogenetic trees clearly showed that the IOLA 16S rRNA genes and IOLA-like sequences were more closely related to eukaryotic mitochondria than typical intracellular bacteria (including *Rickettsiales* bacteria), and some endosymbiotic bacteria (Figure 2 and Figure S2). Though *Candidatus* Carsonella ruddii [26], a endosymbiont of *Pachypsylla venusta*, was classified into *Gammaproteobacteria* (class), the 16S rRNA gene sequences of *Candidatus* Carsonella ruddii were also located in a eukaryotic mitochondria cluster with the IOLA 16S rRNA genes. The results suggested that IOLA might be either a mitochondrion of eukaryotic organism or a unique endosymbiotic bacterium. We hypothesized that the size, shape, and genomic feature of IOLA differs from bacterial cells (if IOLA is a eukaryotic cell).

### Size determination of IOLA

**Figure 3:**
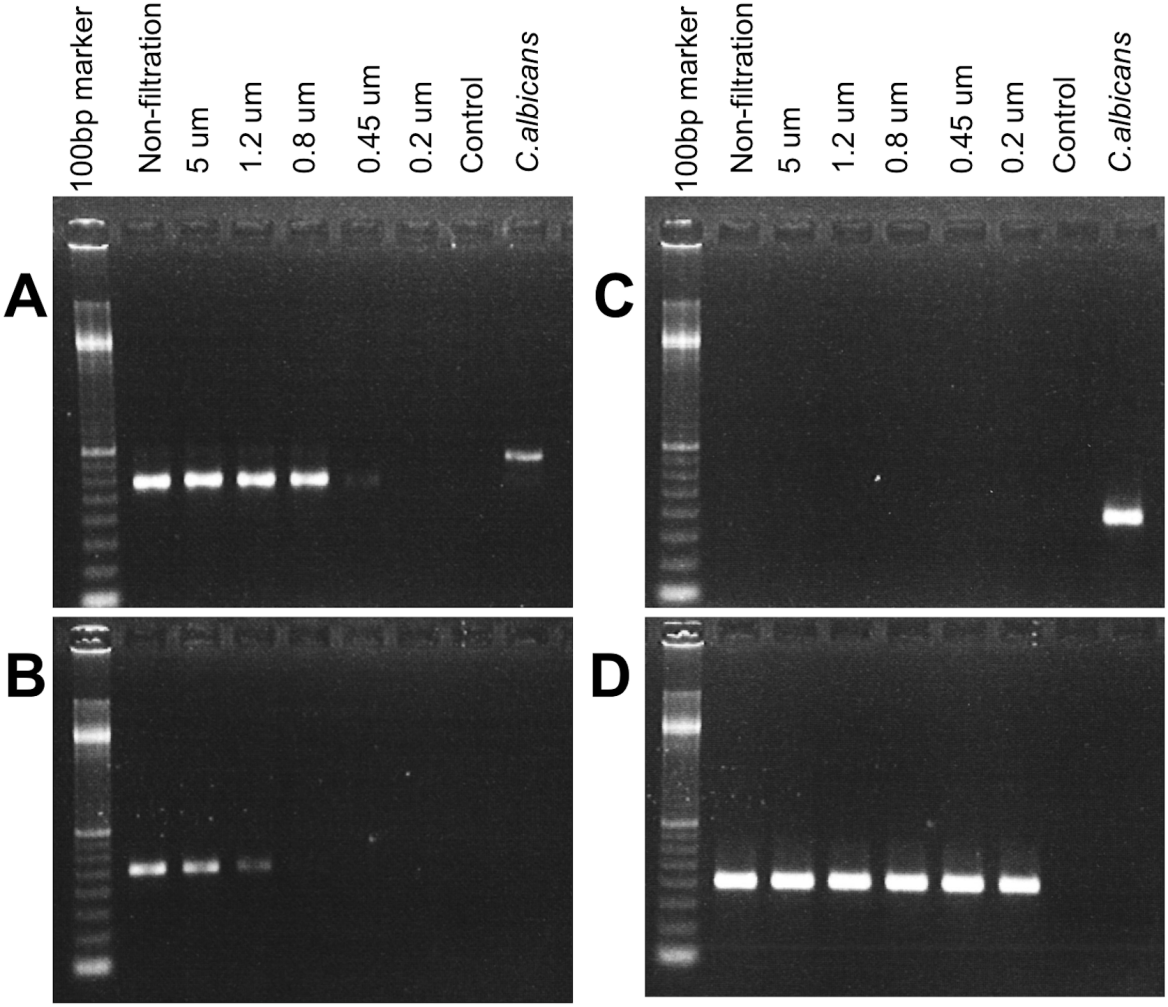
Size estimation of the IOLA cells by filtration and assessment of 18S rRNA gene. In order to estimate the size of IOLA, the PCR examinations were conducted by extracting and purifying the DNAs from the filtrates series of the BALF (A3). **A,** PCR results using a bacterial universal primer set (E341F and E907R). **B,** PCR results using an IOLA-specific primer set (IOLA-F1N and IOLA-R0N). **C,** PCR results using a primer set (18S_0067a_deg and NSR 399) for 18S rRNA genes of various eukaryotic groups. **D,** PCR results using a primer set (Human-P and NSR 399) for human 18S rRNA gene. “Control” indicates no template control reaction. The DNA extracted from *Candida albicans* was used as a positive/negative reaction control of the PCRs for 18S rRNA gene.

To estimate the size of IOLA, the A4 BALF specimen was filtered by cellulose acetate filter with different pore sizes (5, 1.2, 0.8, 0.45, and 0.2 µm), and the filtrate including IOLA was evaluated with bacterial universal PCR and IOLA-specific PCR. Using the bacterial universal primer set, significant PCR results were observed in the filtrates of more than 0.8 µm (Figure 3A). On the other hand, IOLA-specific PCR showed significant PCR results in the filtrates of more than 1.2 µm (Figure 3B). These results suggested that the size of IOLA was approximately 1 µm, and equivalent to small bacteria rather than typical eukaryotic cells. If IOLA is a eukaryote, IOLA must possess 18S rRNA gene(s). However, no significant PCR amplicon was observed from *all* of the filtrates using a primer set for 18S rRNA genes [27] of various eukaryotic groups (excluding human18S rRNA genes) (Figure 3C), remarkably, the amplicon using a specific primer set for human 18S rRNA genes [27] was detected from all of the filtrates (Fig. 3D). Although it is not enough to confirm that IOLA does not possess the 18S rRNA gene, the results have raised the prospect that IOLA is not a eukaryotic cell.

### Fluorescence In Situ Hybridization (FISH) analysis

**Figure 4:**
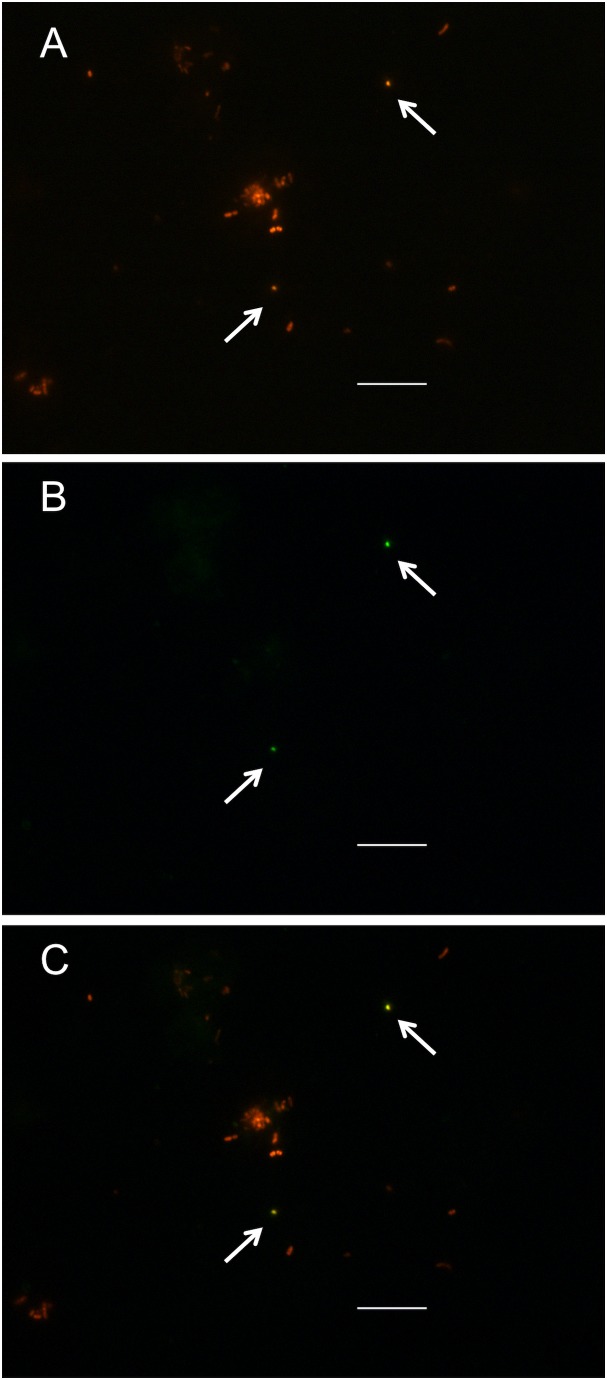
Determination of the size and shape of IOLA in the BALF specimen with FISH micrographs. **A,** A3 BALF stained with the Eub342 probe (red; Cy-3). **B,** The same section as in **A,** stained with the SP0N probe (green; FITC). **C,** The same section as in **A** and **B** stained with Eub342 and SP0N probes. The scale bars indicate 10 µm. The arrows indicate the probable IOLA objects.

To visualize the shape and the state of IOLA in BALF, we performed FISH examinations. With 3 out of the 4 IOLA specific probes (Table S2), plural positive objects were detected in both A3 and A4 specimens (Figure 4 and Figure S3). In A3, which was determined to be a mixed infectious specimen (Figure 1), various size and shape objects were observed with the Eub 342 probe (Figure 4A), and small spherical (or elliptical) objects were observed with both the Eub 342 probe and the IOLA specific probe (Figure 4A–4C), Small spherical objects were also detected in the A4 specimen (Figure S3D–S3F). The objects with approximately 1 µm in diameter were equivalent to the estimated size of IOLA with the filtrate examinations. Under microscopic observation, the spherical objects were scattered independently in some fields and no densely gathering images of these objects were found with both A3 and A4 specimens. The size, shape, and state of IOLA help speculate that IOLA may be a type of bacteria.

### IOLA genomic fragment analysis

If IOLA is a eukaryotic mitochondrion, some features of mitochondrial genome should be observed in the genome of IOLA, therefore, we amplified the genomic fragment including 16S rRNA gene of IOLA with a unique method using single primer PCR and Phi29 DNA polymerase (Table S3–S4, and Figure S4–S5). A total of 18,834 bp of genomic fragment sequence (accession No. AB828323) of IOLA was determined. The homology results from NCBI blastn and genomic BLAST (used blastn and megablast) against all datasets, including Microbial (5,751 genomes), Fungi (296 genomes), Plants (87,456 sequences), Nematodes (771,854 sequences), Environmental (15,049,531 meta-genomic sequences), Protozoa (4,312,506 genomic sequences), and Eukaryota (442 genomes), showed that there is no sequence significantly similar to the IOLA genomic fragment in the datasets available on NCBI website.

**Figure 5:**
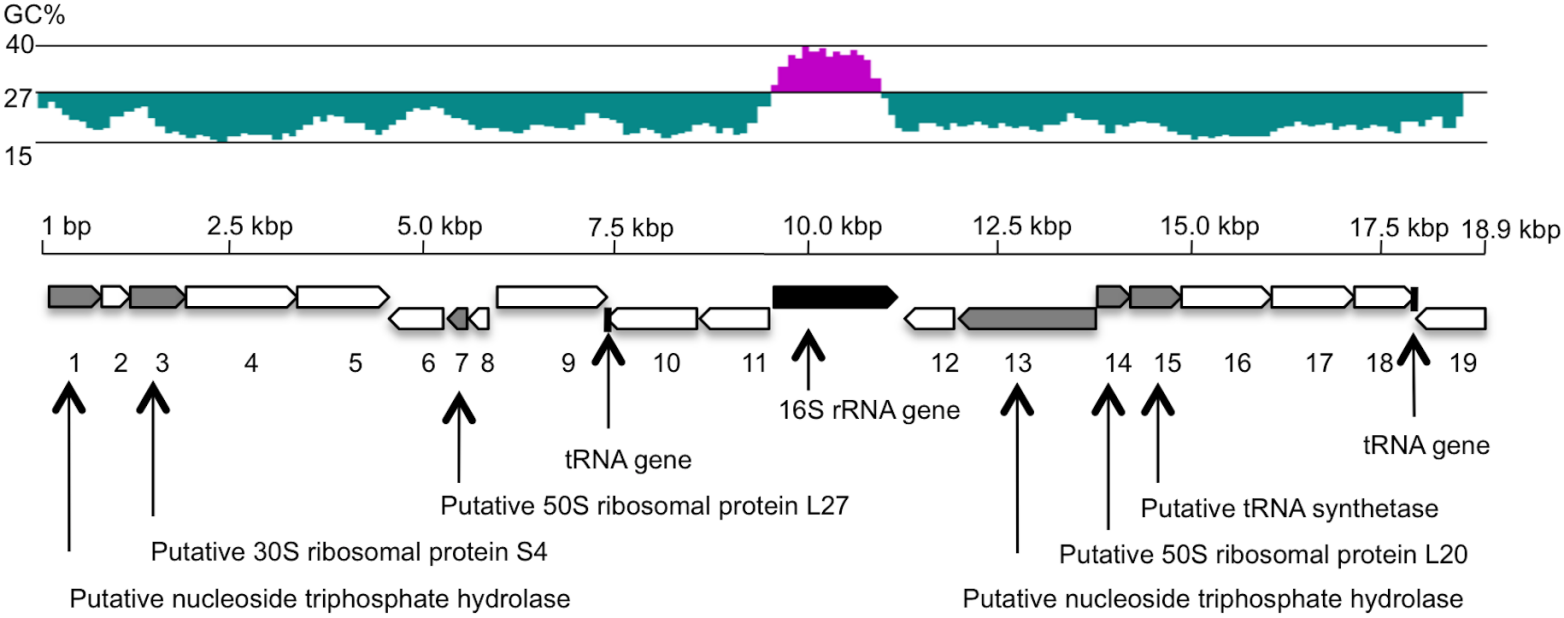
GC content and annotation of IOLA genomic fragment (18,834 bp). High GC contents per 500-base pairs (kbp). The black box represents a 16S rRNA gene. The other boxes represent ORFs. The shaded boxes show similarities (amino-acid sequence) with known bacterial proteins. Annotation results of the ORFs are indicated via arrows. White boxes represent products of ORFs showing extremely low similarities with known proteins.

Except for a 16S rRNA gene, 19 predicted protein-coding sequences (CDSs) and 2 tRNA genes were defined by the annotation using Microbial Genome Annotation Pipeline on DDBJ (http://migap.ddbj.nig.ac.jp). The genes lined up closely (the CDSs and RNA genes cover 95.9% of the 18,834 bp sequence). The G+C ratio of the 18,834 bp was 20.5%. A relatively high G+C ratio was observed specifically within the 16S rRNA gene sequence (Figure 5), however, GC content of IOLA-16S rRNA gene (37.2%) was lower than typical bacteria (for example, *E. coli* K12: 54.6%). This is equivalent to the primary endosymbionts of psyllids (GC contents of their 16S rRNA genes: 35 to 38%) [26]. The genomic features of IOLA such as a high gene density and an extremely low GC content are similar to bacterial endosymbionts such as *Candidatus* Carsonella ruddii (the CDSs and RNA genes cover 97.3% of the genome, and the GC content is 16.5%) [28]. Most of the amino-acid sequences of the CDSs showed insignificant similarities with known proteins (Table 2). Only 6 of the 19 CDSs were (barely) assignable from the NCBI Protein BLAST (blastp) results (Figure 5 and Table 2). All of those 6 known proteins have been found on the genomes of 6 different respective bacteria. Moreover, 2 of 3 ribosomal proteins found in those 6 CDSs were similar to bacterial specific ribosomal proteins (50S ribosomal protein L27 and 50S ribosomal protein L20) [29]. However, the 2 tRNA genes found on the IOLA fragment were similar to mitochondrial tRNA genes; the genes showed 82% similarity with tRNA-Ala of *Huperzia squarrosa* mitochondrion, and 85% with tRNA-Phe of *Chlorella* sp. ArM0029B mitochondrion, respectively (Table 2).

**Table 2:** Blast search results of the CDSs on IOLA genomic fragment.

CDSs	Size (aa)	Predicted Function(conserved domain)	Blast hit {size} (organism)	Identities	Positives	Coverage	E-value	Accession No.
ORF1	220	NTPase (ParA, Fer4_NifH**)**	chromosome partitioning proteinParA {221 aa} (*Helicobacter bilis*)	44/169 (26%)	88/169 (52%)	72%	8.00E-05	WP_005220018
ORF2	146	hypothetical protein	hypothetical protein {541aa}(*Fibrobacter succinogene*)	41/133 (31%)	57/133 (42%)	86%	1	YP_003248808
ORF3	207	30S ribosomal protein S4(rpsD)	30S ribosomal protein S4 {204 aa}(*Oceanibaculum indicum*)	61/168 (36%)	87/168 (51%)	80%	9.00E-17	WP_008944522
ORF4	484	hypothetical protein	arginyl-tRNA synthetase {568 aa}(*Marinitoga piezophila*)	90/307 (29%)	145/307 (47%)	59%	4.00E-22	YP_005096202
ORF5	405	hypothetical protein	uncharacterized protein {1408 aa}(*Oryzias latipes*)	64/266 (24%)	117/266 (43%)	60%	2.8	XP_004080895
ORF6	255	hypothetical protein	GTPase {333 aa}(SAR86 cluster bacterium SAR86A)	51/142 (36%)	72/142 (50%)	50%	1.00E-04	EJP71581
ORF7	82	50S ribosomal protein L27(rpmA)	50S ribosomal protein L27 {85 aa}(*Sulcia muelleri* CARI)	51/82 (62%)	62/82 (75%)	98%	2.00E-22	YP_003880684
ORF8	100	hypothetical protein	hypothetical protein {381 aa}(*Talaromyces stipitatus*)	20/73 (27%)	42/73 (57%)	73%	0.31	XP_002486341
ORF9	496	hypothetical protein	hypothetical protein {411 aa}(*Moraxella boevrei*)	51/185 (28%)	82/185 (44%)	35%	3.3	WP_019520390
tRNA	73(base)	tRNA-Ala	tRNA-Ala {73 base}(*Huperzia squarrosa* mitochondrion)	60/73 (82%)	-	100%	2.00E-09	JQ002659
ORF10	352	hypothetical protein	No significant similarity found	-	-	-	-	-
ORF11	296	Hypothetical protein	No significant similarity found	-	-	-	-	-
rRNA	1504(base)	16S ribosomal RNA	16S ribosomal RNA {767 base}(uncultured Coxiellaceae bacteriumclone BL009B59)	765/767 (99%)	-	50%	0.0	AY806120
ORF12	210	hypothetical protein	hypothetical protein {222 aa}(*Pseudomonas* sp. GM25)	23/81 (28%)	39/81 (48%)	37%	7.2	WP_007955624
ORF13	592	NTPase(lepA, Ras_like_GTPase)	GTP-binding protein LepA {597 aa}(*Desulfobulbus propionicus*)	164/605 (27%)	289/605 (47%)	97%	7.00E-54	YP_004195254
ORF14	117	50S ribosomal protein L20(Ribosomal_L20)	ribosomal protein L20 {117 aa}(*Clostridium papyrosolvens*)	46/107 (43%)	65/107 (60%)	91%	2.00E-17	EGD48990
ORF15	220	tRNA synthetase (PheS)	tRNA synthetase {338 aa}(*Psychromonas ingrahamii*)	68/220 (31%)	113/220 (51%)	96%	9.00E-31	YP_942819
ORF16	383	hypothetical protein	No significant similarity found	-	-	-	-	-
ORF17	340	hypothetical protein	Chromosome partition protein {1181 aa}(*Thermobrachium celere*)	57/187 (30%)	85/187 (45%)	51%	5.6	WP_018661516
ORF18	262	hypothetical protein	Chromosome partition protein {1110aa}(*Clostridium bartlettii*)	50/131 (38%)	76/131 (58%)	47%	4.1	WP_007287303
tRNA	72(base)	tRNA-Phe	tRNA-Phe {73 base}(*Chlorella* sp. ArM0029B mitochondrion)	60/71 (85%)	-	97%	5.00E-11	KF554428
ORF19	277	hypothetical protein	replication factor C subunit 2 {228 aa}(*Nematocida* sp. 1 ERTm2)	42/136 (31%)	72/136 (52%)	48%	1.00E-12	EHY66666

## Discussion

We already confirmed that (1) bacterial cells were uncountable by the epifluorescent microscopic analysis (under the detection limit) and (2) PCR amplifications of the 16S rRNA gene were not observed in the BALFs obtained from 30 patients with idiopathic interstitial pneumonias as “noninfectious controls” [3]. According to our previous report [3], we believe that bacterial infectious pneumonia is distinguishable from pneumonia with noninfectious causes by the bacteria cell counts in BALF specimens. In a previous detailed study of bacterial populations in the healthy human respiratory tract, it was also demonstrated that bacteria are present in the lungs of healthy humans at low levels, and have a similar community composition to the upper airway microbiome [8]. We therefore speculate that the IOLA is an infectious microorganism in human airways based on the following 3 points. First, the IOLA clones were predominantly detected only in specimens from patients with lower respiratory tract infections such as bacterial pneumonia and bronchiectasis. Second, a large number of bacterial cells (>10^6^ cells/ml) were observed in the BALF specimens. Third, clones very similar to the sequence of IOLA had also been detected in BALF specimens in patients with CF, bronchial asthma and COPD in other studies from 2 other countries [12], [20]. The authors, who reported uncultured *Coxiellaceae* bacteria (potential IOLA-like organisms) detected in BALF specimens obtained from CF patients in the USA, surmised that there might be more infectious episodes caused by the parasitic bacteria which were undetectable by ordinary cultivation methods [20]. The number of IOLA-like clones detected in patients with COPD in the UK [12] may not be a significant part of their study; there were no descriptions of IOLA-like organisms in the article. In the process of IOLA-like 16S rRNA gene assessment, we found IOLA-like genes, despite extremely low compositions (0.02% and 0.2%), also in 2 of 50 meta-genomic datasets obtained from throat swabs of non-infectious wheezing infants and health infants, there were no descriptions of IOLA-like organisms [9]. It is possible that IOLA-like organisms inhabit minor-populations in healthy lungs, however, we feel that it is unlikely to be inhabiting major-populations in healthy lungs. The uncovered facts suggest that (1) IOLA exist in the air and (2) IOLA colonize in the lower respiratory tract via human airways.

All phylogenetic analyses in this study revealed that the 16S rRNA gene sequence of IOLA was more similar to mitochondrial 16S rRNA genes of eukaryotic organisms than known bacteria including candidates of mitochondrial ancestor. On the other hand, the phylogenetic analyses showed the possibility of IOLA being a unique endosymbiot, such as *Candidatus* Carsonella ruddii. Morever, the results of filtration study, FISH examinations, and the genomic fragment analysis strongly supported the speculation that IOLA is a type of bacteria. There are no genes related to the electron transport chain in the IOLA genomic fragment (18,834 bp), which is equivalent in length to mitochondrial genomes of most metazoa cells [30], though mitochondrial genomes usually possess many genes encoding the proteins related to the electron transport chain [30].

Interestingly, although 16S rRNA genes are usually located in rRNA operon with other subunits (5S and 23S) in many bacterial genomes, neither the 5S nor the 23S rRNA genes were found near the 16S rRNA gene of IOLA. The genomes of *Rickettsiales* bacteria [31]–[36] have already been reported to have single rRNA operon with the 16S rRNA separated by several hundred kb from the 23S-5S genes, and our results indicate that the gene orientation of IOLA may resemble genomes of *Rickettsiales* bacteria. The detection of CDSs similar to bacterial specific ribosomal proteins on the IOLA genomic fragment also supports the hypothesis that IOLA is a type of bacteria. Contrarily, the phylogenetic analyses of 16S rRNA genes and the homology results of the tRNA gene sequences suggest that IOLA may be a eukaryotic cell mitochondrion. Currently, it is impossible to determine whether IOLA is a type of bacteria or if it is an unknown eukaryote. However, the results obtained so far are proof that IOLA is a novel bacteria-like microorganism possessing the features of eukaryotic mitochondria.

One or more mismatched nucleotides against the bacterial universal primers (27f, E341F, 530f, 1100r, 1392r, 1492r and 1525r) [37] were detected on the IOLA-16S rRNA gene sequences. The fact that most sequences of the universal primers were not completely identical with the sequences of IOLA may propose a serious problem for researchers. We were probably able to detect the 16S rRNA gene sequence of IOLA because the E341F and E907R primers barely matched the sequence of 16S rRNA genes of IOLA. We found a total of 16 IOLA-like 16S rRNA gene sequences in public databases. All of the IOLA-like sequences were amplified using the primer sets corresponding to E341F (or 27f) and E907R [9], [12], [20]. It is suspected that primer mismatch may cause PCR bias, potentially obstructing the detection of IOLA. The whole meta-genome analysis, which is not limited by a target gene, seems to be a suitable method for the detection of unique pathogens from clinical specimens. However the PCR used to construct libraries in the process of next-generation DNA sequencing [1] might be an obstacle for detecting organisms with extremely low GC contents [38] similar to IOLA. The peculiarity of the 16S rRNA gene and the genome (extremely low GC contents) might be an obstacle for detecting organisms similar to IOLA. In our study, conventional culture-based methods could not detect IOLA. If IOLA is an obligate endosymbiotic organism, it may be difficult (perhaps impossible) to detect IOLA by culture-based methods. Moreover, being unculturable and having unique molecular biological characteristics may be the principal reasons for the latency of IOLA in human airways. The results in our study suggest that we may be able to see a wider range of microorganisms than previously believed, by improvements in primer sequence and PCR conditions.

Considering all the results, two main conclusions were reached: (1) IOLA is a novel bacteria-like microorganism also possessing the features of eukaryotic mitochondria. Although there is an on going heated debate about the lineage between bacteria and mitochondria [31], [39], [40], IOLA seems to be a significant organism for understanding the origins and history of mitochondria. (2) IOLA is a candidate for a novel pathogen, which may or may not be one of the causative agents in human lung or airway diseases. We speculate that IOLA may be an airborne organism, and that IOLA-like “unknown microorganisms” may exist worldwide. Our results provide evidence that unknown microorganism(s), which may be difficult to detect even using molecular methods, exist in the human body. Furthermore, our results may help to discover other human-based yet-to-be-found microorganisms.

## Materials and Methods

### Subjects

Six clinical specimens were obtained from 3 patients in our university hospital and referred hospitals between September 2010 and June 2012. This study was approved by the Human and Animal Ethics Review Committee of the University of Occupational and Environmental Health, Japan (No.09-118). Written informed consent was obtained from the patients. BALF specimens were collected according to a previous report [3].

### Bacterial cell counts and bacterial flora analysis

To evaluate the bacterial flora in the clinical specimens, we performed the total bacterial cell counts and the 16S rRNA gene clone library analysis, as previously reported [3], [4].

### Culture examinations

In order to culture IOLA, A4 BALF specimen (less than 24 hours after BAL examination) was used. The specimen was stored at 4°C until applying to cultivation. Twelve agar mediums, a semisolid medium, and 2 kinds of liquid mediums were used (Table S1). The aliquots (50 µl) of the BALF specimen diluted 10-fold with PBS were spread on each of the 12 kinds of agar plates. The dilutions (10 µl) were injected into the liquid mediums. As for the semisolid medium (GAM Semisolid), the BALF specimen was directly stabbed into it with an inoculating needle. The media were incubated under the 3 conditions (aerobic, semianaerobic, and anaerobic) at 30°C and 37°C (two groups) from 24 hours to 2 weeks. Because of the difficulty of discovery of IOLA, 1 ml of distilled water was added to each of the agar plates after the cultivation, and the appearing colonies on the plates were mixed with a bacterial spreader. The DNA were extracted and purified from 50 µl of the bacterial cell suspensions, as previously reported [3], [4]. The same process was applied to the cultivated liquid mediums and the semisolid medium. In order to check for the growth of IOLA, an IOLA-specific PCR was conducted. Two specific primers (IOLA-F1; 5′-AGTGATGAAGGCATTAACTTG-3′ and IOLA-R0; 5′-GCTTAGACATTAGCTAAACAC-3′) for IOLA 16S rRNA gene were designed referring to the 550 bp clone sequence, and the specificities of primers were assessed with BLASTn. The 25 µl PCR reaction mixtures contained 100 nM of each of the primers (IOLA-F1 and IOLA-R0), 1 µl of the extracted DNA and AmpliTaq Gold DNA polymerase were incubated at 96°C for 5 min, followed by 30 cycles at 96°C for 30 sec, at 53°C for 30 sec, at 72°C for 1 min, and then 1 cycle for the final elongation step at 72°C for 2 min. The PCR amplicon was evaluated with 2% agarose gel electrophoresis.

Infection experiments using a J774 murine macrophage-like cell line and an A549 human adenocarcinoma cell line were performed, as previously reported [41], [42]. The 100 µl of BALF specimen (A4) was inoculated onto A549 and J774 cells in each well. After the infected cells were cultured for 3 to 6 days, the growth of IOLA in both the culture mediums and the infected cells were checked with the IOLA-specific PCR.

### Determination of IOLA 16S rRNA gene sequences

An approximation of the full length of the IOLA 16S rRNA gene was determined by assembling the sequences of 2 PCR amplicons (Figure S1). The DNAs extracted from A1, A4, B1, and C1 BALF specimens as described [3], [4] were used as templates for the each of the PCR reactions. The reaction mixtures contained the primer set [27f and IOLA-R0, or IOLA-F1 and 1492r] and AmpliTaq Gold DNA polymerase (Applied Biosystems) were incubated at 96°C for 5 min, followed by 30 cycles at 96°C for 30 sec, at 55°C for 30 sec, at 72°C for 2 min, and then 1 cycle for the final elongation step at 72°C for 2 min. After treatment with an ExoSAP-IT (GE Health care UK Ltd.), 1 µl of PCR mixture was used as a template for the sequencing reaction. The sequencing reactions were performed with the primer sets (for the amplicon of 27f and IOLA-R0; 27f, E341F, 519r, and IOLA-R0, for the amplicon of IOLA-F1 and 1492r, IOLA-F1, 926f, 1100r, and 1492r) and BigDye Terminator Cycle Sequencing Kit version 3.1 (Applied Biosystems). The nucleic acid sequences were determined with a 3130xl Genetic Analyzer (Applied Biosystems). The sequences were assembled with DNASIS-Pro software (Hitachi Solutions, Ltd.), and then 1,427 bp of IOLA 16S rRNA gene sequences were determined.

### Analyses of IOLA 16S rRNA gene sequences

The IOLA-16S rRNA gene sequences (1,427 bp) were compared with “nr” database and sequence read archive database using the NCBI blastn. In order to locate IOLA-like organisms, we used the Classifier of the RDP II. To obtain the rough phylogenic information of IOLA 16S rRNA gene sequences, phylogenetic analysis was performed with ARB-software and the SSU Ref NR 111 dataset including 286,858 sequences of ribosomal small subunit of Eucarya, Archaea, and Bacteria.

More detailed phylogenetic trees were constructed using MEGA5.2.2 software. The data set of 16S rRNA gene sequences (71 sequences) was composed of as follows: 1 chloroplast (used as an outgroup), 1 *Escherichia coli* (strain K12), 11 typical intracellular bacteria (*Chlamydia* spp., *Parachlamydia acanthamoebae*, *Mycoplasma* spp., *Legionella* spp., *Coxiella burnetii*, *Brucella* spp., and *Salmonella enterica*), 11 *Rickettsiales* bacteria (*Rickettsia* spp., *Orientia tsutsugamushi*, *Anaplasma* spp., *Ehrlichia* spp., and *Candidatus* Midichloria spp.), 2 *Pelagibacter* (SAR11 clade), 9 endosymbiotic or symbiotic bacteria, 24 mitochondria of eukaryotic organisms, 8 IOLA-like uncultured bacteria sequences, and 4 IOLA (Figure 2 and Figure S2). The sequences, except for 8 IOLA-like sequences, were standardized in a length (1,320 base). The data set was aligned using Clustal W with the default setting in MEGA5.2.2 software. For ML, the model of nucleotide substitutions was selected with the “Find Best DNA/Protein Models (ML)” tool of MEGA5.2.2 software. An ML tree was constructed using the General Time Reversible substitution model [43] with gamma distribution and proportions of invariable sites. A neighbor-joining tree was constructed using the Kimura 2-parameter model [44]. For both trees, gaps were treated with partial deletion, and node support was estimated using 1000 bootstrap replicates.

### Size determination of IOLA

The filtrates of BALF (A3) were prepared by using cellulose acetate filter with different pore sizes (5, 1.2, 0.8, 0.45, and 0.2 µm) (Sartorius). The diluted BALF solution was prepared by adding 3,250 µl of PBS to 250 µl of the fresh A3 BALF (less than 2 hours after BAL examination). An aliquot (300 µl) of the solution was transferred into a new tube. The remaining 3,200 µl was filtrated with 5 µm filter, and then an aliquot (300 µl) of the filtrate was transferred into a different fresh tube. The remaining filtrate was filtrated with the next size (1.2 µm) filter, and then an aliquot (300 µl) of the filtrate was transferred into a different fresh tube. This filtration process (with decreasing pore sizes) was repeated 3 times, and then, 5 filtrates (300 µl of each) were obtained in the end. The DNA was extracted from each filtrate by in the same method as BALF. The size of the IOLA was estimated by the amplicons of PCR examinations using a specific primer set (IOLA-F1 and IOLA-R0) in the same conditions described previously (culture examination section). PCR using the bacterial universal primer set (E341F and E907R) was also performed with the same conditions. To determine the existence of eukaryotic cells, the PCR using the primer sets for 18S rRNA genes of various eukaryotic groups (18S_0067a_deg and NSR 399) and for human 18S rRNA gene (Human-P; 5′-AAGCCA TRCATGTCTAAGTACGC-3′ and NSR 399) were also performed as described in a previous report [27].

### FISH analysis

A fresh A4 BALF sample (less than 3 hours after BAL examination) and an A3 BALF sample (stored with 40% glycerol at −80°C for about 2 months) were used for FISH analysis. The BALF specimens (50 µl) were added to 950 µl of 4% paraformaldehyde, and were incubated at 4°C overnight. The bacterial cells that were fixed in the mixed solution (total 1 ml) were collected on a 25-mm-diameter polytetrafluoroethylene (PTFE) filter (pore size, 0.2 µm) (Advantec). Then, the PTFE filter was washed with 1 ml of 50% ethanol, and then was dried on a paper towel. Probes labeled at 5′-end with fluorescein-5- isothiocyanate (FITC) for IOLA were designed from the 16S rRNA gene sequence, and specificities of the probe sequences (Table S2) were assessed with the NCBI blastn. The bacterial universal probes Eub342, labeled at 5′-end with Cy3 were used to detect bacterial cells in the BALF specimens. Hybridization buffer (900 mM NaCl, 20 mM Tris-HCl [pH 7.5], 35% formamide, 0.01% SDS, 50 nM a universal probe, and 50 nM an IOLA-specific probe) and washing buffer (80 mM NaCl, 20 mM Tris-HCl [pH 7.5], 5 mM EDTA, 0.01% SDS) were prepared in accord to previous reports [45], [46]. The fixed bacterial cells on the PTFE filter were soaked in 2 ml of the hybridization buffer in a 35-mm-diameter polystyrene culture dish (Corning). After covering with Parafilm (Pechiney Plastic Packaging Company), the hybridization mixture in the dish was incubated at 46°C for 3 hrs in a multi-shaker oven HB (Taitec). The filter was removed from the dish and washed twice in a fresh dish containing 2 ml of washing buffer at 46°C for 15 min. After the filter was dried on a paper towel, the filter was put onto a slide glass, mounted with 10 µl of the antiquenching reagent solution (200 mM DABCO, 20 mM Tris-HCl [pH 7.5], 90% Glycerol) and a cover glass. Digital images of the slides, observed with an ECLIPSE Ni-U microscope (Nikon), were taken with a DS-Fi2 digital camera (Nikon).

### IOLA genomic fragment analysis

A partial DNA including 16S rRNA gene of IOLA was amplified by using the single primer PCR and Phi29 DNA polymerase from the DNA extracted from the A4 BALF specimen (Figure S4, Table S3–S4). The DNA was amplified with an Illustra Genomiphi DNA Amplification Kit (GE healthcare life sciences), according to the manufacturer’s instructions. The Genomiphi reaction mix was diluted 10-fold with TE buffer. The first single-strand DNAs were prepared by using KOD FX Neo DNA polymerase (Toyobo) and the newly designed IOLA-specific primers (Table S3, and Figure S4A). The 50 µl of reaction mixture [containing 25 µl of 2×PCR buffer for KOD FX Neo, 10 µl of dNTPs (2 mM), 1 µl (10 pmol) of an IOLA-specific primer (IOLA-RGAM F1 or IOLA-RGAM R1), 12 µl of ultra-pure water, 1 µl of KOD FX Neo DNA polymerase (1U), and 1 µl of the diluted genomiphi reaction mix (as template DNA)] was incubated in a thermocycler (GeneAmp 9700) at 94°C for 2 min, followed by 25 cycles at 94°C for 30 sec, at 68°C for 9 min. After the reaction, the reaction mixture was diluted 20-fold with TE buffer. In order to amplify double-strands DNA from the first single-strand DNA of IOLA, an Illustra genomiphi DNA Amplification Kit was used again. After the 1 µl of diluted first single-strand DNA solution was mixed with 9 µl of sample buffer, skipping over only the heat denature step, the genomiphi reaction protocol was continued according to the manufacturer’s instructions (Figure S4B). The genomiphi reaction mixture was diluted 20-fold with TE buffer, and then, it was used for the second single-strand DNA preparation. In the process, the reaction conditions were the same as in the first single-strand DNAs preparation, but only the primer was different. Each primer (IOLA-RGAM F2 or IOLA-RGAM R2) used in the second single-strand DNA preparation was located at about 400 bases downstream of the each primer (IOLA-RGAM F1 or IOLA-RGAM R1) (Table S3). When the products were analyzed by 1% agarose gel electrophoresis, a smear (approximately over 5 kbp) was observed in both forward (F) and reverse (R) cases (Figure S4C). The unusual products (the smear) were purified by using the Wizard DNA Clean-Up System (Promega). Approximately 10 µg of the purified products were sheared and cloned by using the TOPO Shotgun Subcloning Kit (Invitrogen). Then, the nucleotide sequences of the clones were determined by using BigDye Terminator Cycle Sequencing Kit and a 3130xl Genetic Analyzer. A total of 192 sequence reads were assembled with a Contig Manager software (Hitachi Solutions, Ltd.). A large contig (10,151 bases composed of 106 sequence reads) was obtained (Figure S4A). The new primers located on both end (5′- and 3′-) of the contig were generated (Table S4), and were used to obtain the sequence information of the outside region of the contig. The same operations described above were additionally executed for both sides of the 10,151 bases contig. Finally, the sequence of 19,305 bases was roughly estimated. The new primers (Table S4) were generated referring to the rough sequence, and used for PCR examinations to confirm the sequence. In order to confirm the sequence, 21 fragments, which overlap with each other on the about 19 kb, were established and amplified with KOD FX Neo DNA polymerase from the original DNA extracted from A4 BALF specimen (Figure S5). The sizes of the amplicons were confirmed by using 1% agarose gel electrophoresis (Figure S5C–S5D). Sequences of the DNA fragments were determined by direct sequencing (Table S4), and then the new sequences were assembled. Finally, the genomic sequence (18,834 bp) including IOLA 16S rRNA gene was determined (Figure S5B). The genomic sequence was assessed with NCBI blastn and genomic BLAST in NCBI website. Annotation of the partial sequence was performed with the Microbial Genome Annotation Pipeline on DDBJ and the NCBI blastp. To extract ORF and RNA regions, the MetaGeneAnnotator 1.0 [47] and tRNAscan-SE 1.23 [48] software were used. Annotation based on the similarities of the amino-acid sequences of each of the defined ORFs was done with the NCBI blastp and the non-redundant protein sequences database (nr). In this study, the confidence threshold values were set as follows: overlap length (>70%), positive identities (>45%).

## Supporting Information

Figure S1 **Strategy of amplification and sequencing of IOLA 16S rRNA gene.** Arrows and broken arrows indicate the location of bacterial universal primers and IOLA-specific primers, respectively. **A**, The partial sequence of IOLA 16S rRNA gene obtained with the clone library analyses. **B**, PCR amplicon using 27f and IOLA-R0N primers. **C**, PCR amplicon using IOLA-F1N and 1492r primers. **D**, An approximation of the full length of the IOLA 16S rRNA gene was determined by assembling the sequences of 2 PCR amplicons (amplicon **B** and **C**).
(TIFF)

Figure S2 **Neighbor-joining phylogenetic tree based on 16S rRNA gene sequences of bacteria and mitochondria of eukaryota.** The phylogenetic tree was calculated with MEGA5.2.2 using the neighbor-joining method. The 16S rRNA gene sequences and the bootstrap replications used in this analysis are same as in Figure 2.
(TIFF)

Figure S3 **FISH micrographs of the BALF specimens.** **A**–**C**, The micrographs show the A3 BALF stained with the Eub342 probe (red; Cy-3) and the IOLA-specific probes (green; FITC); SP0N, SP2N, and SP3N, respectively. **D**–**F**, The micrographs show the A4 BALF stained with the Eub342 probe and the IOLA-specific probes; SP0N, SP2N, and SP3N, respectively. The scale bars indicate 10 µm. The arrows indicate the probable IOLA objects.
(TIFF)

Figure S4 **Schematic representation of the unique strategy for amplification and sequencing of IOLA genomic fragment. A**, The results of the 1^st^ PCR using single primers (IOLA-RGAM F1 or IOLA-RGAM R1). **B**, The results of the genomiphi reaction to synthesize double-strand DNA from the products of 1^st^ PCR using single primers. **C**, The results of the 2^nd^ PCR using single primers (IOLA-RGAM F2 or IOLA-RGAM R2). Unusual PCR products (smear, over 5 kbp approximately) are observed. The broken black lines represent 1^st^ single primer PCR products. The broken red lines represent 2^nd^ single primer PCR products (unusual PCR products). The shotgun library of the artificial products were prepared with a TOPO shotgun subcloning kit. And then, the sequences of the clones were determined with Sanger method.
(TIFF)

Figure S5 **Confirmation of the IOLA genomic fragment by the size of PCR amplicons.** White boxes indicate the location of the IOLA 16S rRNA gene. The broken lines are the expanded genome legions of IOLA with the single-primer PCR and the genomiphi reactions. Bars with alphabets show the location and size (bp) of PCR amplicons. **A**, The IOLA genomic fragment obtained by assembling the clone sequences (first extension). **B**, The IOLA genomic fragment finally determined (18,834 bp). **C**, **D**, The results of agarose gel electrophoresis analyses of the PCR amplicons. The partial genome sequence (18,834 bp) was determined by the size and the sequencing results of the amplicons (**C** and **D**).
(TIFF)

Table S1 **Culture mediums used to detect IOLA.**
(DOCX)

Table S2 **Oligonucleotide probe sequences used in FISH analyses.**
(DOCX)

Table S3 **Oligonucreotide sequences of primers used to the single primer PCR.**
(DOCX)

Table S4 **Oligonucreotide sequences of primers used for PCR and re-sequencing of the IOLA genomic fragment.**
(DOCX)
