# Supplementary figures and images for: An Unclassified Microorganism: Novel Pathogen Candidate Lurking in Human Airways
Source: PLoS One. 2014 Jul 31;9(7):e103646. doi: 10.1371/journal.pone.0103646 (PMC4117515; doi:10.1371/journal.pone.0103646)

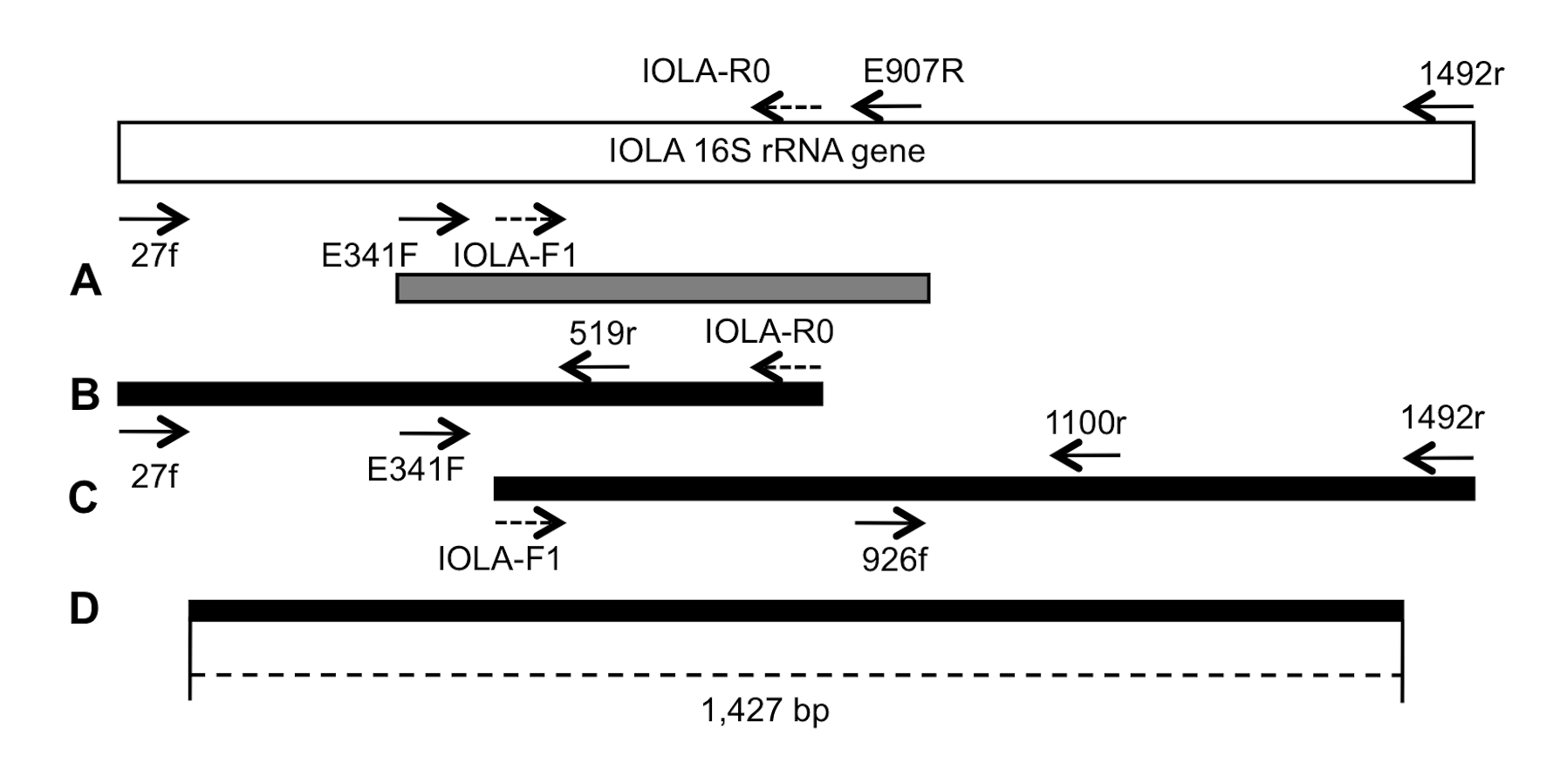

Supplement: Figure S1 — Strategy of amplification and sequencing of IOLA 16S rRNA gene. Arrows and broken arrows indicate the location of bacterial universal primers and IOLA-specific primers, respectively. A, The partial sequence of IOLA 16S rRNA gene obtained with the clone library analyses. B, PCR amplicon using 27f and IOLA-R0N primers. C, PCR amplicon using IOLA-F1N and 1492r primers. D, An approximation of the full length of the IOLA 16S rRNA gene was determined by assembling the sequences of 2 PCR amplicons (amplicon B and C). (TIFF) [file pone.0103646.s001.tiff]

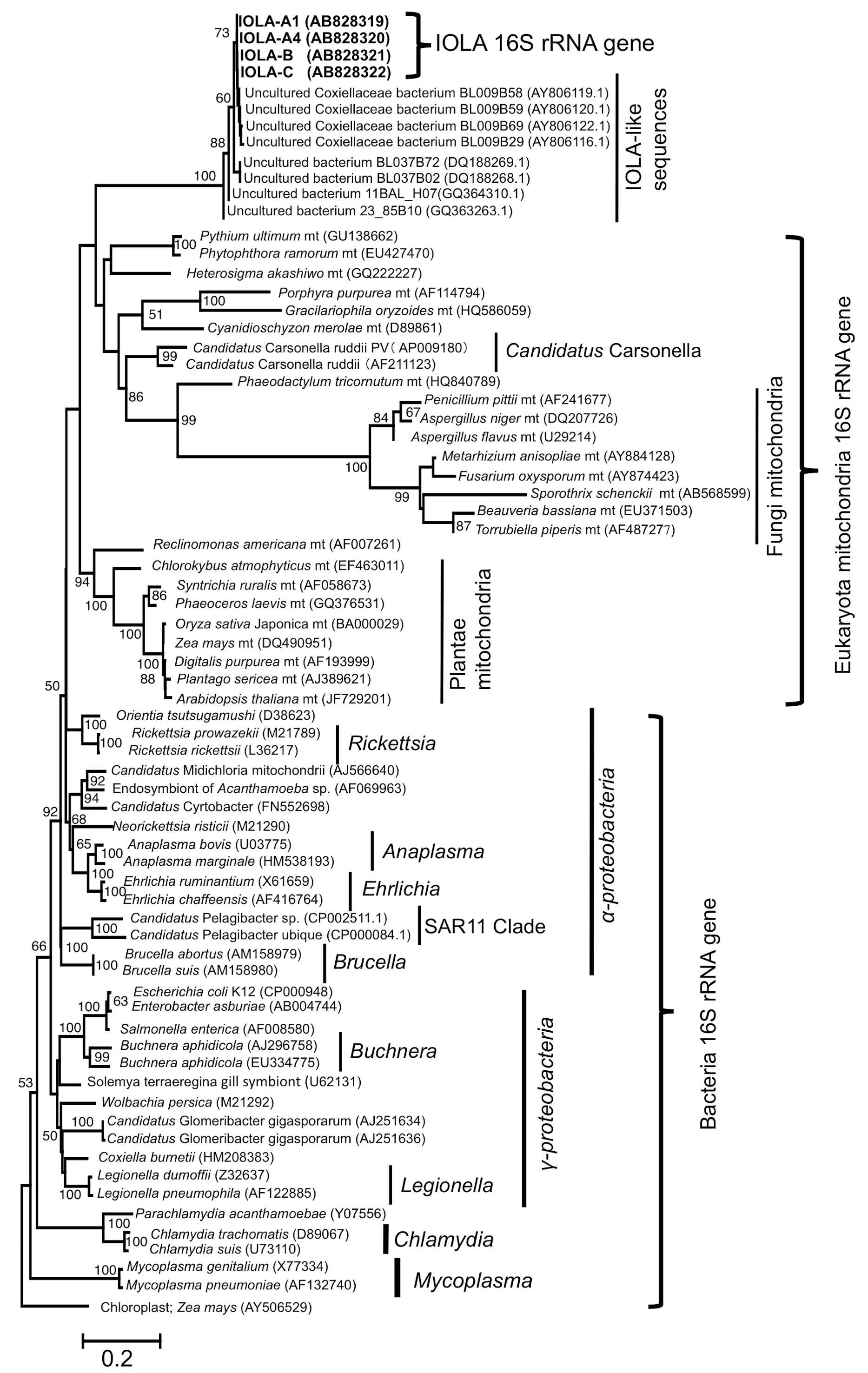

Supplement: Figure S2 — Neighbor-joining phylogenetic tree based on 16S rRNA gene sequences of bacteria and mitochondria of eukaryota. The phylogenetic tree was calculated with MEGA5.2.2 using the neighbor-joining method. The 16S rRNA gene sequences and the bootstrap replications used in this analysis are same as in Figure 2. (TIFF) [file pone.0103646.s002.tiff]

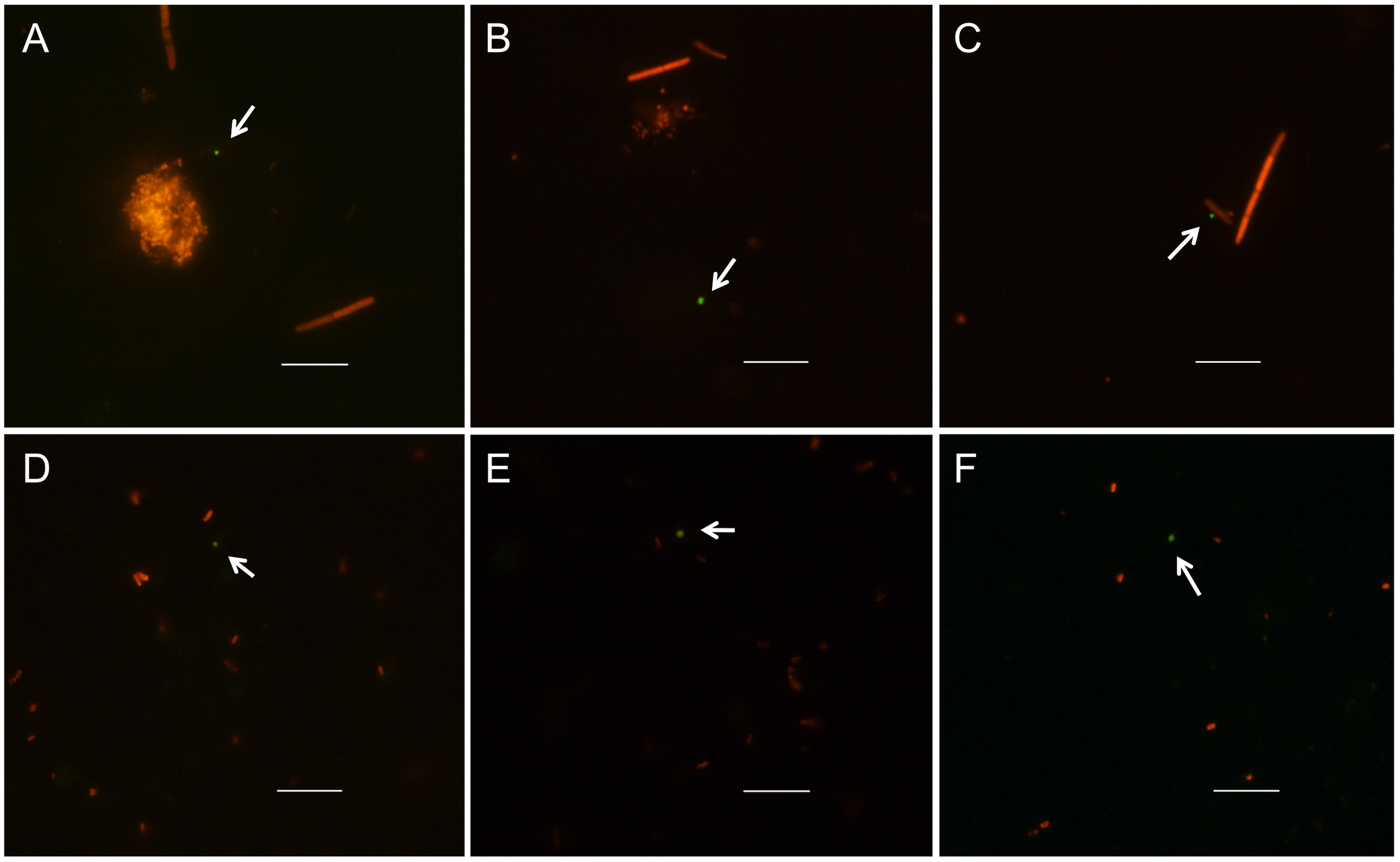

Supplement: Figure S3 — FISH micrographs of the BALF specimens. A–C, The micrographs show the A3 BALF stained with the Eub342 probe (red; Cy-3) and the IOLA-specific probes (green; FITC); SP0N, SP2N, and SP3N, respectively. D–F, The micrographs show the A4 BALF stained with the Eub342 probe and the IOLA-specific probes; SP0N, SP2N, and SP3N, respectively. The scale bars indicate 10 µm. The arrows indicate the probable IOLA objects. (TIFF) [file pone.0103646.s003.tiff]

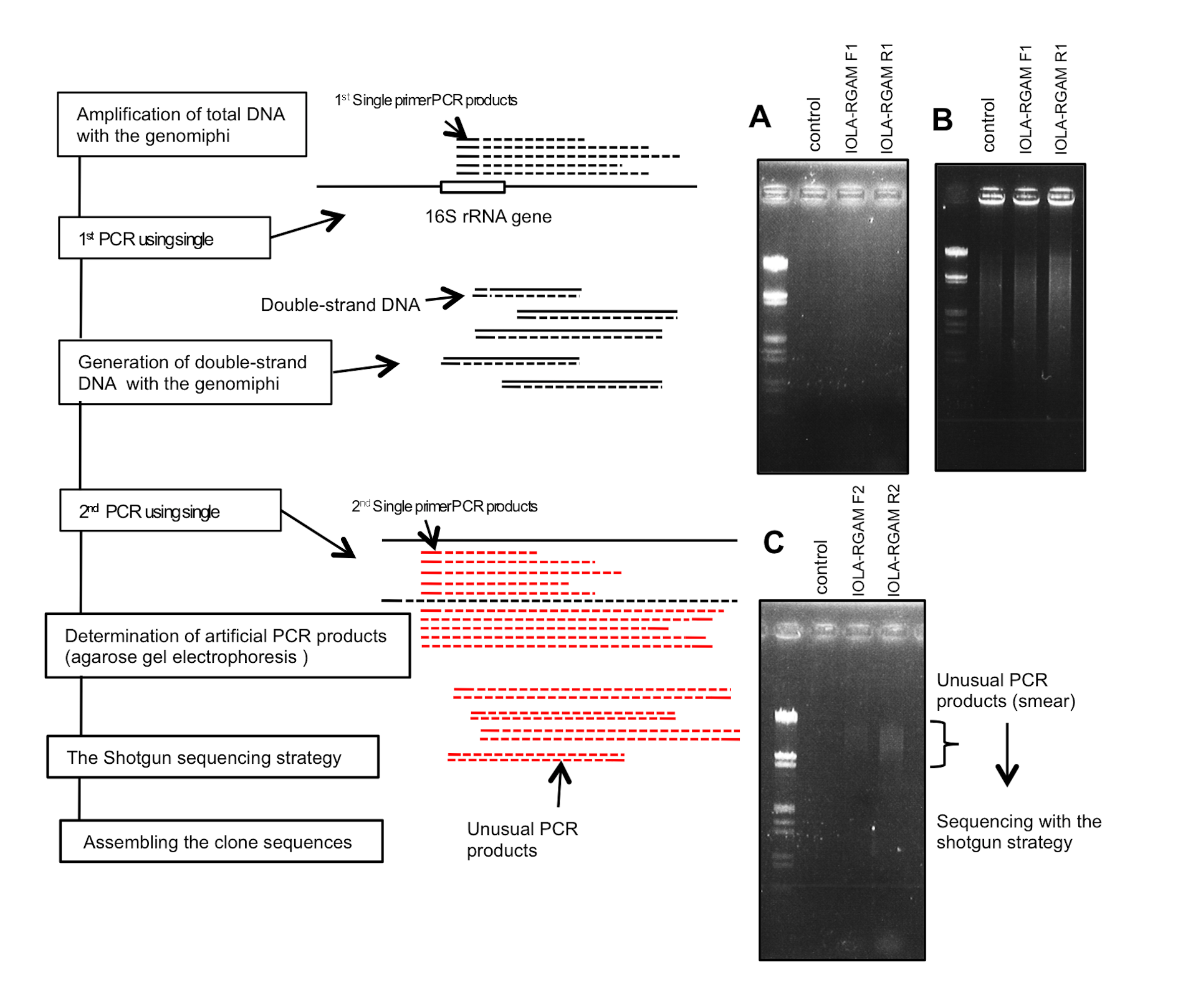

Supplement: Figure S4 — Schematic representation of the unique strategy for amplification and sequencing of IOLA genomic fragment. A, The results of the 1st PCR using single primers (IOLA-RGAM F1 or IOLA-RGAM R1). B, The results of the genomiphi reaction to synthesize double-strand DNA from the products of 1st PCR using single primers. C, The results of the 2nd PCR using single primers (IOLA-RGAM F2 or IOLA-RGAM R2). Unusual PCR products (smear, over 5 kbp approximately) are observed. The broken black lines represent 1st single primer PCR products. The broken red lines represent 2nd single primer PCR products (unusual PCR products). The shotgun library of the artificial products were prepared with a TOPO shotgun subcloning kit. And then, the sequences of the clones were determined with Sanger method. (TIFF) [file pone.0103646.s004.tiff]

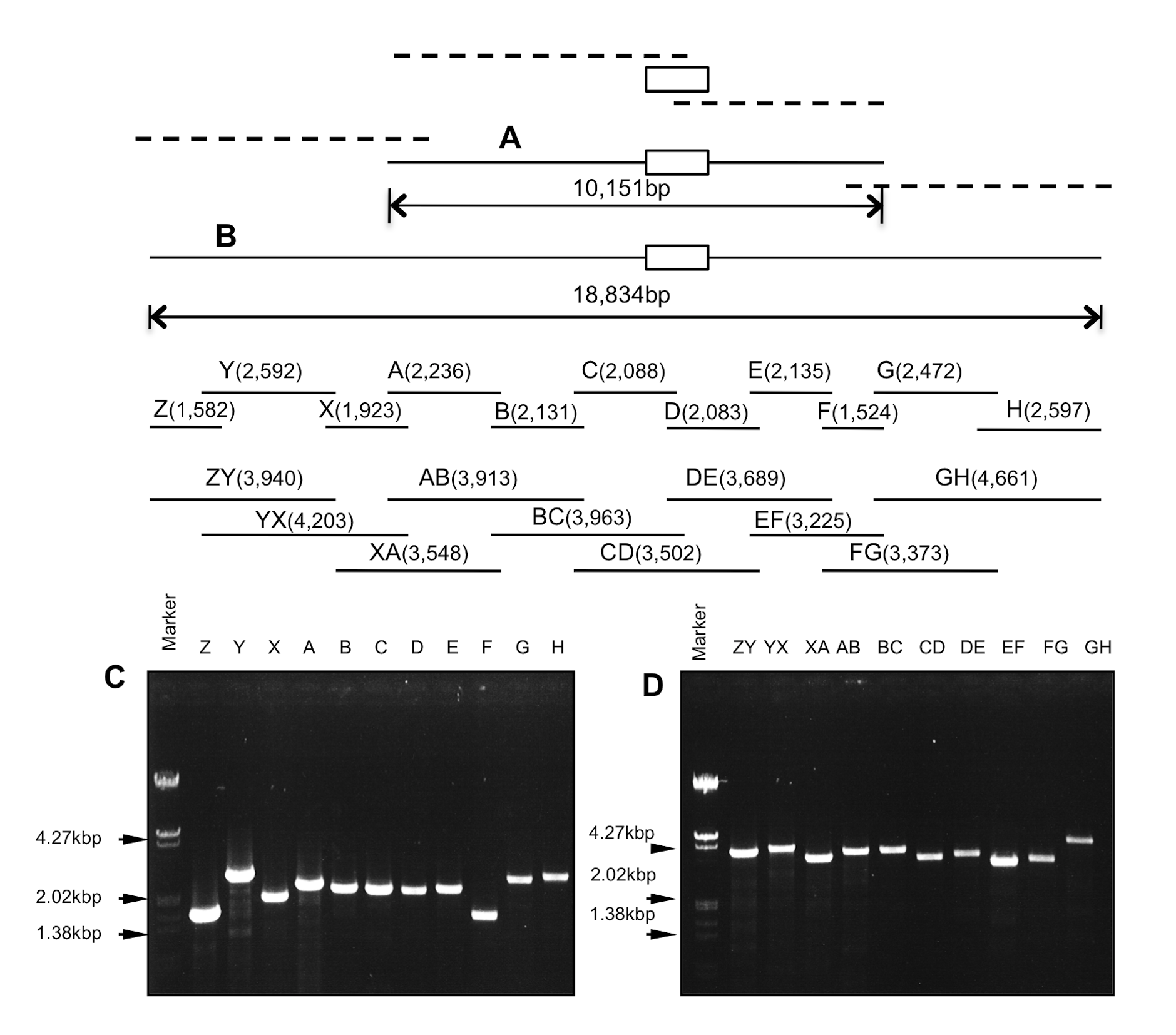

Supplement: Figure S5 — Confirmation of the IOLA genomic fragment by the size of PCR amplicons. White boxes indicate the location of the IOLA 16S rRNA gene. The broken lines are the expanded genome legions of IOLA with the single-primer PCR and the genomiphi reactions. Bars with alphabets show the location and size (bp) of PCR amplicons. A, The IOLA genomic fragment obtained by assembling the clone sequences (first extension). B, The IOLA genomic fragment finally determined (18,834 bp). C, D, The results of agarose gel electrophoresis analyses of the PCR amplicons. The partial genome sequence (18,834 bp) was determined by the size and the sequencing results of the amplicons (C and D). (TIFF) [file pone.0103646.s005.tiff]
