# Supplementary material for: An Unclassified Microorganism: Novel Pathogen Candidate Lurking in Human Airways
Source: PLoS One. 2014 Jul 31;9(7):e103646. doi: 10.1371/journal.pone.0103646 (PMC4117515; doi:10.1371/journal.pone.0103646)
Supplement: Table S1 — Culture mediums used to detect IOLA. (DOCX) [file pone.0103646.s006.docx]

**Table S1. Culture medium used to detect IOLA**

| State (agar conc.) | Medium | Purchased from |
| --- | --- | --- |
| Agar plate  (1.5 %) | Brain Heart Infusion | Becton, Dickinson and Company |
|  | Mueller Hinton Broth | Becton, Dickinson and Company |
|  | Mycobacteria 7H11 Agar | Becton, Dickinson and Company |
|  | Brucella HK Agar | Kyokuto Pharmaceutical Industrial Co. ltd. |
|  | Nutrient Agar | Nissui Pharmaceutical Industrial Co. ltd. |
|  | Sheep Blood Agar | Nissui Pharmaceutical Industrial Co. ltd. |
|  | GAM Broth | Nissui Pharmaceutical Industrial Co. ltd. |
|  | DNA Agar | Nissui Pharmaceutical Industrial Co. ltd. |
|  | RPMI 1640 medium (5% FCS) | Nissui Pharmaceutical Industrial Co. ltd. |
|  | Sabouraud Dextrose Agar | Eiken Chemical Co. ltd. |
|  | SCDLP Agar | Eiken Chemical Co. ltd. |
|  | WYO-α Agar | Eiken Chemical Co. ltd. |
| Semisolid  (0.15 %) | GAM Semisolid | Nissui Pharmaceutical Industrial Co. ltd. |
| Liquid  (0 %) | RPMI 1640 medium (10% FCS) | Nissui Pharmaceutical Industrial Co. ltd. |
|  | FCS | Gibco® |

GAM; Gifu Anaerobic Medium, DNA; Deoxyribonucleic acid, RPMI; Roswell Park Memorial Institute, SCDLP; Soybean-Casein-Digest-Lecithin-Polysorbate80,

WYO; Wadowsky-Yee-Okuda, FCS; Fetal Calf Serum
