# Supplementary material for: An Unclassified Microorganism: Novel Pathogen Candidate Lurking in Human Airways
Source: PLoS One. 2014 Jul 31;9(7):e103646. doi: 10.1371/journal.pone.0103646 (PMC4117515; doi:10.1371/journal.pone.0103646)
Supplement: Table S2 — Oligonucleotide probe sequences used in FISH analyses. (DOCX) [file pone.0103646.s007.docx]

| Probe | 5'-label | Sequence | Length | Target |
| --- | --- | --- | --- | --- |
| Eub 342 | Cy3 | 5’-CTGCTGCCTCCCGTAGG-3’ | 17 mer | Most eubacteria (16S rRNA gene) |
| SF0N | FITC | 5’-GCGATCGTACTACTCAGGTG-3’ | 20 mer | IOLA (16S rRNA gene) |
| SF1N | FITC | 5’-CAAGTTAATGCCTTCATCACTCA-3’ | 23 mer | IOLA (16S rRNA gene) |
| SF2N | FITC | 5’-GCGTGGATTACAAGGGTCTC-3’ | 20 mer | IOLA (16S rRNA gene) |
| SF3N | FITC | 5’-TTGTCTCACCGCGGTTGCTG-3’ | 20 mer | IOLA (16S rRNA gene) |

**Table S2. Oligonucleotide probe sequences used in FISH analyses**
