# Supplementary material for: An Unclassified Microorganism: Novel Pathogen Candidate Lurking in Human Airways
Source: PLoS One. 2014 Jul 31;9(7):e103646. doi: 10.1371/journal.pone.0103646 (PMC4117515; doi:10.1371/journal.pone.0103646)
Supplement: Table S3 — Oligonucreotide sequences of primers used to the single primer PCR. (DOCX) [file pone.0103646.s008.docx]

**Table S3. Oligonucreotide sequences of primers used to the single primer PCR**

| Primer | Purpose | Sequence (5'-3') | Tm (℃） |
| --- | --- | --- | --- |
| IOLA-RGAM F1 | 1st single primer PCR (to extend 3' ) | AGGTGACTTACTCCATGAACAATG | 57.1 |
| IOLA-RGAM F2 | 2nd single primer PCR (to extend 3' ) | GATGAAGGCATTAACTTGTTGTAAAACTC | 57.8 |
| IOLA-RGAM R1 | 1st single primer PCR (to extend 5' ) | CAGAACTCAATAACGTATTCACCG | 57.1 |
| IOLA-RGAM R2 | 2nd single primer PCR (to extend 5' ) | AAGAACTGAATCATACGTCTCACG | 57.1 |
| IOLA-RGAM F3 | 1st single primer PCR (to extend 3' ) | TGAGACAACATCCAGTTTATCGTAC | 57.2 |
| IOLA-RGAM F4 | 2nd single primer PCR (to extend 3' ) | GGACTAGGAATTGAGCGAATAGC | 58.7 |
| IOLA-RGAM R3 | 1st single primer PCR (to extend 5' ) | GAAGAGATAGTATAGGTAGAAGACTTG | 57.5 |
| IOLA-RGAM R4 | 2nd single primer PCR (to extend 5' ) | GAGCTGATACAATAATACATACTTCACAG | 57.8 |
