# Supplementary material for: An Unclassified Microorganism: Novel Pathogen Candidate Lurking in Human Airways
Source: PLoS One. 2014 Jul 31;9(7):e103646. doi: 10.1371/journal.pone.0103646 (PMC4117515; doi:10.1371/journal.pone.0103646)
Supplement: Table S4 — Oligonucreotide sequences of primers used for PCR and re-sequencing of the IOLA genomic fragment. (DOCX) [file pone.0103646.s009.docx]

| Primer | Purpose | Sequence (5'-3') |
| --- | --- | --- |
| Z-F | Fragment Z or ZY PCR and sequencing | ATACCACTGACATTTTGAACATAACC |
| Z-R | Fragment Z PCR and sequencing | CTCTTATAGTTTTAAAGCAACCAGATC |
| Y-F | Fragment Y or YX PCR and sequencing | GACCAACAAGACCTGGTATGCATG |
| Y-R | Fragment Y or ZY PCR and sequencing | TACACTGCTATATAGGACAATATCAG |
| X-F | Fragment X or XA PCR and sequencing | CCAATGATATTAAGATGTAGAGAAATAC |
| X-R | Fragment X or YX PCR and sequencing | GAAGAGATAGTATAGGTAGAAGACTTG |
| A-F | Fragment A or AB PCR and sequencing | TAGCATATCTAGATCCAACTCCTC |
| A-R | Fragment A or XA PCR and sequencing | TACTGGCAATGATGATAGAGTAGC |
| B-F | Fragment B or AB PCR and sequencing | CACATAGTATGTGTGTAATAGATGG |
| B-R | Fragment B or BC PCR and sequencing | CATCTGACATATACAATGGTCATAAG |
| C-F | Fragment C or CD PCR and sequencing | GATACAAGCACCAATTCATTGGAC |
| C-R | Fragment C or BC PCR and sequencing | AAGAACTGAATCATACGTCTCACG |
| D-F | Fragment D or DE PCR and sequencing | GATGAAGGCATTAACTTGTTGTAAAACTC |
| D-R | Fragment D or CD PCR and sequencing | GTAAATGTTATGGTGGAGATAGAAC |
| E-F | Fragment E or EF PCR and sequencing | CACTTAATCCTGCTGAATATGCAAC |
| E-R | Fragment E or DE PCR and sequencing | CATGGTAAATCTACATTTTGCGATG |
| F-F | Fragment F or FG PCR and sequencing | GTCTTAGCCGATATTTCAAATATATCC |
| F-R | Fragment F or EF PCR and sequencing | GCTATTCGCTCAATTCCTAGTCC |
| G-F | Fragment G or GH PCR and sequencing | TGAGACAACATCCAGTTTATCGTAC |
| G-R | Fragment G or FG PCR and sequencing | CAAGATTCATATTTTCTGTTGCAGAAATAG |
| H-F | Fragment H PCR and sequencing | CAGGTGGAGAGAGATTTTATTCTTC |
| H-R | Fragment H or GH PCR and sequencing | GCTGATTTAATATTTCAACTTTGTAGCTC |

**Table S4. Oligonucreotide sequences of primers used for PCR and re-sequencing of**

**the genomic fragment of IOLA**

**Table S4. Continue**

| Primer | Purpose | Sequence (5'-3') |
| --- | --- | --- |
| Z-MF1 | Sequencing | GTCATAACTTCAATGCAGCTTTTATAAC |
| Z-MR1 | Sequencing | CATGCATACCAGGTCTTGTTGGTC |
| Z-MR2 | Sequencing | GTATGTATAGGTGGTATTGGCAC |
| Y-MF1 | Sequencing | GGTAAAATAGTTCATTTTGGGCATTTAAG |
| Y-MF2 | Sequencing | GTATACAGAATCAAAATACTATAGTCTTTG |
| Y-MR1 | Sequencing | CCAATTTTATCTAATAAGTTCTCAAGATTC |
| Y-MR2 | Sequencing | CAAAGACTATAGTATTTTGATTCTGTATAC |
| X-MF1 | Sequencing | GTTTAGCAGCATTTTGATATCCATATTAG |
| X-MR1 | Sequencing | GAGCTGATACAATAATACATACTTCACAG |
| X-MR2 | Sequencing | CCTTATAGCTTTCTAACTAATTCTATAC |
| A-MF1 | Sequencing | GAAAATATCCTATATGCTGATTATATC |
| A-MF2 | Sequencing | CTCTCAGATTATTATAGATAATCTTGAAAGAG |
| A-MR1 | Sequencing | CCATCTATTACACACATACTATGTG |
| A-MR2 | Sequencing | GCTGTTAATTAGACTATTCTCAAG |
| B-MF1 | Sequencing | AGACGATCGGAATCGAACCGAC |
| B-MF2 | Sequencing | GGGTCTTTTTGAGTATATTCATGTTG |
| B-MR1 | Sequencing | CCAATACAAATTTCTTTATTATCACATCC |
| C-MF1 | Sequencing | TTGTTTCAAAAATACAGACTTTACACC |
| C-MF2 | Sequencing | AGGTGACTTACTCCATGAACAATG |
| C-MR1 | Sequencing | CTTCCATCAATCTCAATTAATACAGTATC |
| C-MR2 | Sequencing | TCATTGTTCATGGAGTAAGTCACC |
| D-MF1 | Sequencing | GTCGTGAGACGTATGATTCAGTTC |
| D-MR1 | Sequencing | GTTGCATATTCAGCAGGATTAAGTG |
| D-MR2 | Sequencing | CAGAACTCAATAACGTATTCACCG |
| E-MF1 | Sequencing | TGCTTCTTCATGTTGTTGGTCAG |
| E-MR1 | Sequencing | GGATATATTTGAAATATCGGCTAAGAC |
| F-MR1 | Sequencing | GATTTTTGAAAACTTGTCGTATGAGTTC |
| G-MF1 | Sequencing | GGACTAGGAATTGAGCGAATAGC |
| G-MF2 | Sequencing | CTGCAAAAGAGATACTAACACATCC |
| G-MR1 | Sequencing | GCCTATTAATCTACAAGCACTG |
| H-MF1 | Sequencing | GAATTAAGGATTAATCTTGCATTACAAG |
| H-MF2 | Sequencing | GATAAAGAAGAGATTAAAAAGTCCTATAGC |
| H-MF3 | Sequencing | CTCTACCACTAATTTTAAAGACCAAAC |
| H-MR1 | Sequencing | TGTTAATTAGACTTTTATCTGCCTAGAG |
| H-MR2 | Sequencing | CTGAGATCAATTGTTACATCTGAAATAC |
